# Supplementary material for: Effect of arbuscular mycorrhizal symbiosis on growth and biochemical characteristics of Chinese fir (Cunninghamia lanceolata) seedlings under low phosphorus environment
Source: PeerJ. 2024 Mar 22;12:e17138. doi: 10.7717/peerj.17138 (PMC10962349; doi:10.7717/peerj.17138)
Supplement: Supplemental Information 1 [file peerj-12-17138-s001.docx]

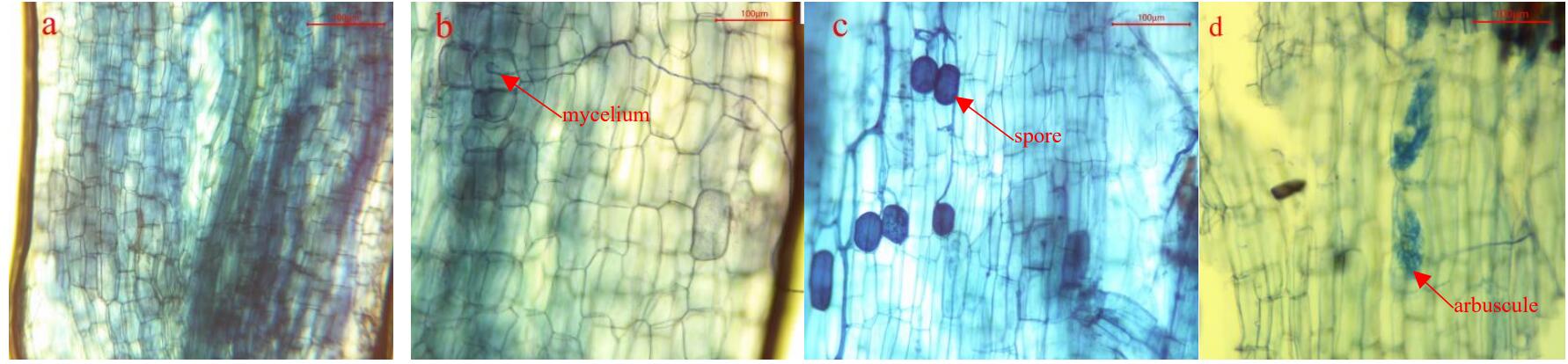


**Figure A1.** A schematic diagram of AMF infection on Chinese fir roots. In this figure, Fig.A1a represents the roots of Chinese fir without AMF inoculation, Fig.A1b-d represents the roots of Chinese fir infected by AMF. The mycelial structure was observed in Fig.A1b, the spore structure in Fig.A1c, and the arbuscule structure in Fig.1d.
